# Retinal Microvasculature in Systemic Sclerosis Patients and the Correlation between Nailfold Capillaroscopic Findings and Optical Coherence Angiography Results

**DOI:** 10.3390/jcm13072025

**Published:** 2024-03-30

**Authors:** Katarzyna Paczwa, Magdalena Rerych, Katarzyna Romanowska-Próchnicka, Marzena Olesińska, Radosław Różycki, Joanna Gołębiewska

**Affiliations:** 1 Department of Ophthalmology, Military Institute of Aviation Medicine, 01-755 Warsaw, Polandmagdalena.rerych@gmail.com (M.R.);; 2 Department and Polyclinic of Systemic Connective Tissue Diseases, National Institute of Geriatrics, Rheumatology and Rehabilitation, 02-637 Warsaw, Poland

**Keywords:** systemic sclerosis, scleroderma, retinal perfusion, nailfold capillaroscopy, optical coherence tomography angiography, OCTA

## Abstract

**Background:** The comparison of retinal perfusion in the eyes of patients with systemic sclerosis (SSc) and in healthy controls using optical coherence tomography angiography (OCTA). The correlation between nailfold capillaroscopy results and OCTA findings among SSc. **Methods:** The study enrolled 31 patients with systemic sclerosis and 41 healthy controls. OCTA was performed in both groups to assess the retinal vasculature in the superficial (SCP) and deep (DCP) capillary plexuses and the foveal avascular zone (FAZ) area. Nailfold capillaroscopy (NC) was performed in SSc patients and compared to the FAZ area and the superficial and the deep vessel density. **Results:** In the SSc group, the parafoveal vessel density in DCP was significantly higher in relation to the mean value (*p* < 0.0001) and in each quadrant of the macula (*p* < 0.0001) compared to healthy subjects (*p* < 0.0001). The patients with early scleroderma patterns in capillaroscopy had a larger superficial and deep FAZ (*p* = 0.0104, *p* = 0.0076, respectively) than those with active and late patterns. There was a statistically significant difference in the FAZ when comparing early to active (*p* < 0.0001) and early to late scleroderma patterns (*p* < 0.0001). A statistically significant difference was found in the type of interstitial lung disease and the deep FAZ area (*p* = 0.0484). SSc patients with nonspecific interstitial pneumonia (NSIP) had a larger FAZ than those with usual interstitial pneumonia (UIP) (*p* = 0.0484). Moreover, NSIP cases had a higher parafoveal mean superficial vessel density than those with UIP (*p* = 0.0471). **Conclusions:** Our investigation showed that the peripheral microvascular system correlates with ocular microcirculatory impairment. The results indicate the important role of OCTA in the diagnosis, monitoring, and prognosis of microvascular changes in SSc.

## 1. Introduction

Systemic sclerosis (SSc) is a rare and chronic autoimmune disease of the connective tissue [1]. It affects about 2.5 million people worldwide, with a female predominance [2]. The disease is divided into two main subtypes: limited cutaneous SSc (lcSSc) and diffuse cutaneous SSc (dcSSc) [3]. SSc manifests through an abnormal immunological response, vasculopathy, and fibrosis, leading to organ dysfunction [1]. The complex interplay of these pathological processes considerably affects vital organs such as the lungs, heart, kidneys, muscles, skin, and eyes [4].

These cumulative effects lead to a quality-of-life deterioration and increased mortality. Microvasculopathy is an important and initial pathogenic link responsible for early SSc manifestations like Raynaud’s phenomenon and changes in nailfold capillaries [5].

Endothelial damage is one of the mechanisms leading to disorders of vascular hemostasis, platelet activation, and thrombosis, causing coagulopathy and microangiopathy.

Moreover, it has been proven that structural changes appear in the microvasculatory system before inflammation and fibrosis and play a crucial role in the diagnostic process and treatment response in those with scleroderma [5,6].

Nailfold capillaroscopy (NC) is a non-invasive tool included in the diagnostic criteria for SSc in the 2013 American Collage of Rheumatology (ACR) and the European League Against Rheumatism (EULAR) guidelines. It is used in everyday practice to evaluate the number, size, and shape of the blood vessels in the nailfold microcirculation [6,7,8].

Currently, changes in NC are categorized using Cutolo et al.’s classification into three patterns: early, active, and late [9]. However, vascular pathologies can occur in other organs, including the eye, especially the retinal microvasculature system.

Optical coherence tomography angiography (OCTA) is a non-invasive imaging method that provides highly detailed, three-dimensional images of the entire microvasculature of the retina and choroid, enabling retinal perfusion assessment. It identifies the retinal vessels by detecting and measuring the movement of the blood cells in them without dye injection [10,11]. A wide variety of ocular manifestations has been reported in SSc patients, including the anterior and posterior segments of the eye [12,13,14,15,16]. However, there are inconsistent data regarding retinal perfusion measured using OCTA in SSc patients compared to capillaroscopy results. 

The aim of our study was to compare the microvasculature changes in patients with SSc to those in healthy volunteers using OCTA and to evaluate the correlation between the peripheral microvascular changes seen in NC and the characteristics of scleroderma and the OCTA results obtained in the study group.

## 2. Materials and Methods

This cross-sectional study was conducted between March 2022 and May 2023 at the Department of Ophthalmology of the Military Institute of Aviation Medicine in Warsaw, Poland. The study was approved by the Ethics Committee of the Military Institute of Aviation Medicine and followed the tenets of the Declaration of Helsinki. The participants signed a written consent form after the detailed explanation of the nature of the study.

The study group consisted of patients with scleroderma from the Department and Polyclinic of Systemic Connective Tissue Diseases of the National Institute of Geriatrics, Rheumatology and Rehabilitation in Warsaw, Poland. Healthy controls were recruited during routine visits to the Ophthalmology Department of the Military Institute of Aviation Medicine.

The exclusion criteria encompassed refractive defects exceeding −3 D and +3 D; the presence of eye diseases such glaucoma, retinal and choroidal pathologies, and uveitis; and low-quality OCTA images. 

**Figure 1:**
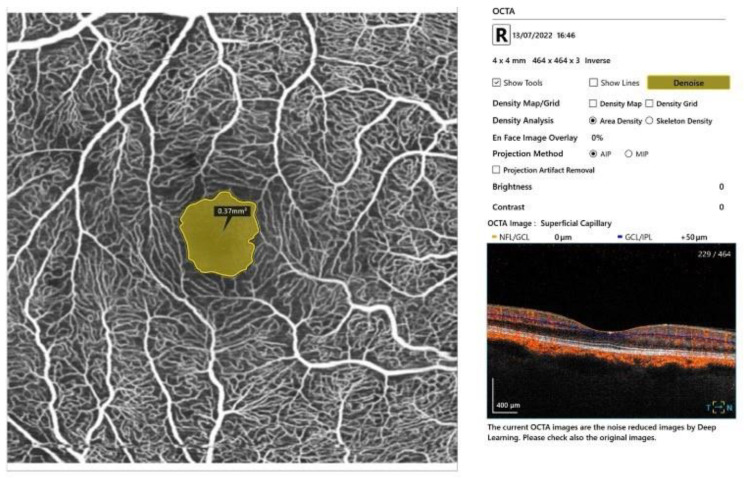
Representative OCTA report of patient with SSc. An image of the macular superficial capillary plexus with the FAZ area measured.

Every participant underwent a complete ophthalmic examination, including a refraction test, best-corrected visual acuity (BCVA), tonometry, segment slit-lamp examination of the anterior segment, and dilated fundus evaluation using a 90-diopter (D) lens. BCVA was measured monocularly using LogMAR charts (Lighthouse International, New York, NY, USA) at a distance of 5 m. OCT and OCTA were performed using SS-OCT (Xephilio^®^ OCT-A1, Canon, Tokyo, Japan). The OCTA scans covered a region of 4 mm × 4 mm. The scan automatically inserted fovea-centered circles at the macula, detecting the superficial capillary plexus (SCP) and the deep capillary plexus (DCP). The SCP network was segmented from 3 μm beneath the internal limiting membrane to 15 μm below the inner plexiform layer (IPL). The DCP was segmented from 15 to 70 μm below the IPL. The foveal vessel density was defined as the area of the small circle, with a diameter of 1 mm. The parafoveal vessel density was defined as the area of the outer circle. It was automatically divided into four quadrants: superior (S), inferior (I), nasal (N), and temporal (T). The vessel density was automatically calculated as a percentage, according to the area occupied by blood vessels, by the software. The average vessel density of the SCP and DCP in the four quadrants was calculated. The foveal avascular zone (FAZ) in the deep and the superficial plexus was measured using the automated procedure provided with the software. Two scans for each eye were captured, and the best one in terms of quality was considered for the analysis (Figure 1).

Moreover, all SSc patients underwent a clinical evaluation, laboratory examination, and nailfold capillaroscopy. The data collected were the type of SSc, i.e., limited or diffuse cutaneous scleroderma; interstitial lung disease defined as nonspecific interstitial pneumonia (NSIP) or usual interstitial pneumonia (UIP); hypertension; elevated proBNP; and the treatment method—mycophenolate mofetil (MMf) or methotrexat (MTX)—as well as sildenafil or amlodipine administration. 

In this study, nailfold capillaroscopy was conducted using the Dino-Lite capillaroscope. The device was set up according to the manufacturer’s guidelines, and participants were positioned to expose the nailfold area for examination. The same expert systematically captured the images, ensuring consistency in lighting, focus, and positioning.

The objective was to classify scleroderma patterns according to the Cutolo classification and assess various parameters, including the vessel number, hemorrhages, branched-shaped vessels, giant capillaries, and avascular areas. This classification was based on the presence of specific capillary abnormalities associated with different disease stages.

The number of vessels detected in each capillaroscopy image was categorized as follows: normal—over 7, reduced—between 6 and 4, and very reduced—3 or fewer capillaries. This classification provided insights into the microvascular changes associated with scleroderma progression.

Hemorrhages, branched-shaped vessels, giant capillaries, and avascular areas were meticulously documented for each participant. These parameters can contribute to a comprehensive understanding of the microvascular abnormalities characteristic of scleroderma [17].

Regular quality control checks were performed on the Dino-Lite capillaroscope, and calibration images were captured to ensure accurate color representation and resolutions.

The study’s endpoint was to evaluate and describe the scleroderma patterns based on the Cutolo classification and the categorized vessel numbers. Additionally, a detailed description of other capillaroscopy parameters, including hemorrhages, branched-shaped vessels, giant capillaries, and avascular areas, was provided.

Another goal was to evaluate the correlation between the nailfold capillaroscopy results and the OCTA parameters in patients with systemic sclerosis. 

The final aim of the study was to compare the OCTA parameters in the study group to those of healthy controls.

### Statistical Analysis

Categorical traits were depicted through integers and percentages. Numerical variables were described by the weighted arithmetic mean, median, standard deviation, and minimum-to-maximum values. 

For contingency tables, a chi-squared test of independency or Fisher’s exact test (for small cell numbers) was applied. The normality of distribution was assessed by using the Shapiro–Wilk W test. The homogeneity of variances was assessed by Levene’s test. An analysis of variance (for normally distributed variables) or generalized linear models (for non-normally distributed ones) were applied to assess differences in numerical variables between the study groups. All models were controlled for age and gender.

Due to the fact that the multifactor analyses encompassed measurements for two eyes per patient, standard error correction consisting of intra-subject correlations was applied. 

In order to illustrate and describe the degree of separability in selected angiological measurements by the prevalence of scleroderma, ROC curves were plotted and corresponding cut-off points were proposed, adding sensitivity and specificity values. In this context, the Youden index was applied.

A level of *p* < 0.05 was deemed statistically significant. All statistical procedures were performed by using STATGRAPHICS Centurion, version 19.4 (Statgraphics Technologies, Inc., The Plains, VA, USA).

## 3. Results

A total of 61 eyes of 31 patients with systemic sclerosis were included in this study and compared to the OCTA data of 81 eyes of 41 healthy controls. There was no significant difference between the scleroderma patients and controls in terms of the age and gender distribution.

In our study group, 71.4% of patients had diffuse cutaneous systemic sclerosis and 28.6% had limited cutaneous SSc. The nailfold capillaroscopy findings in this group revealed 5 patients with an early pattern, 12 patients with an active pattern, and 9 patients with a late pattern. Among the SSc patients, only one had pulmonary arterial hypertension (PAH), eight subjects had finger ulcers, and eight suffered from hypertension. A total of 13 participants were treated with sildenafil and 14 with amlodipide. Interstitial lung disease was observed in 12 patients (67% NSIP and 33% UIP). Elevated proBNP was detected in 17.8% of the study group (Table 1 and Table 2).

There was no statistically significant difference in the superficial vessel density (SVD) or the FAZ measurements between the study group and the controls. However, the parafoveal deep vessel density (DVD) was significantly higher in SSc patients than in control subjects, considering both its mean value (*p* < 0.0001) and every quadrant: superior, inferior, temporal, and nasal (respectively, *p* < 0.0001) (Table 3). 

**Figure 2:**
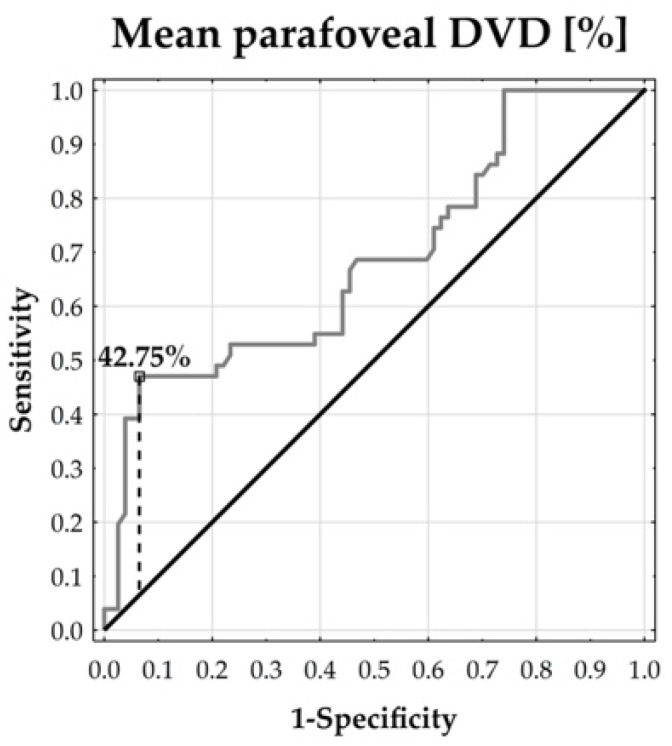
ROC curve for mean parafoveal DVD. AUC = 68.49% (*p* = 0.0002).

**Figure 3:**
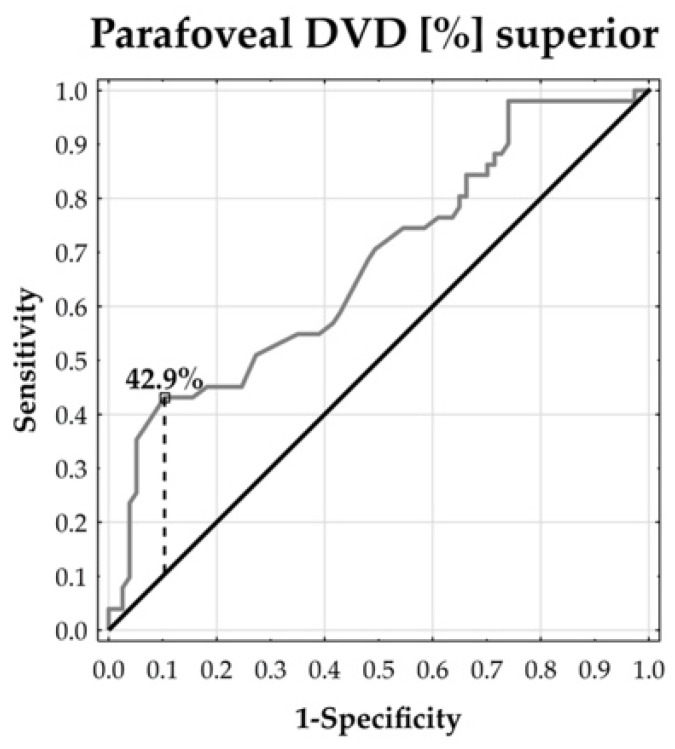
ROC curve for superior DVD. AUC = 67.71% (*p* = 0.0003).

**Figure 4:**
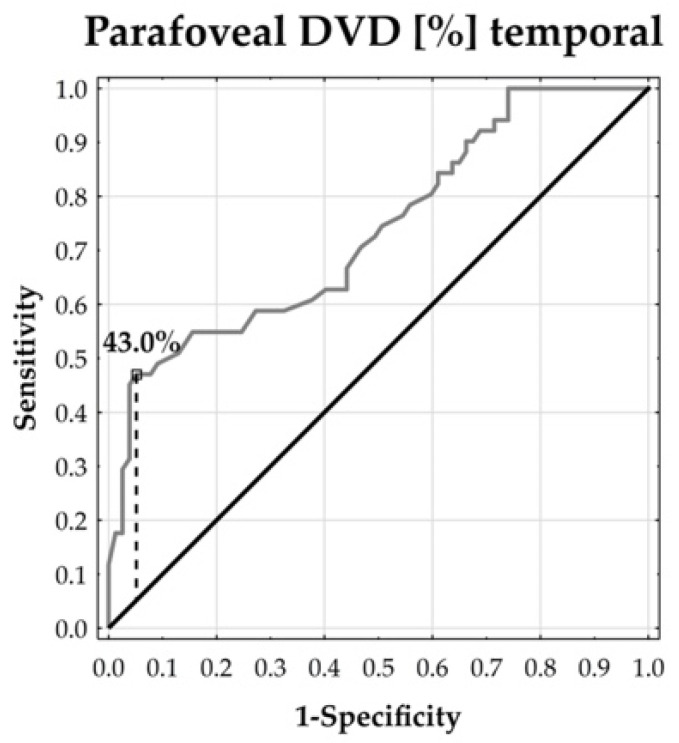
ROC curve for temporal DVD. AUC = 73.57% (*p* < 0.0001).

**Figure 5:**
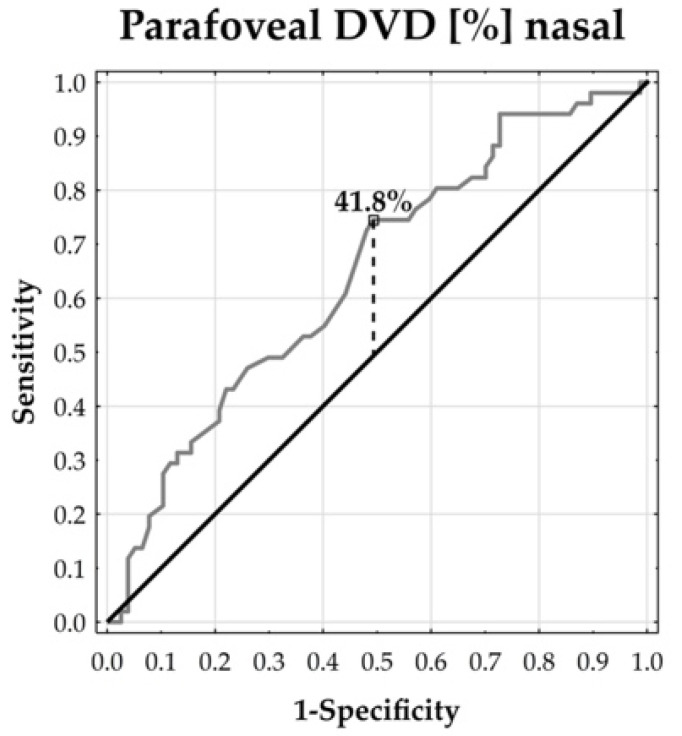
ROC curve for nasal DVD. AUC = 64.46% (*p* = 0.0034).

**Figure 6:**
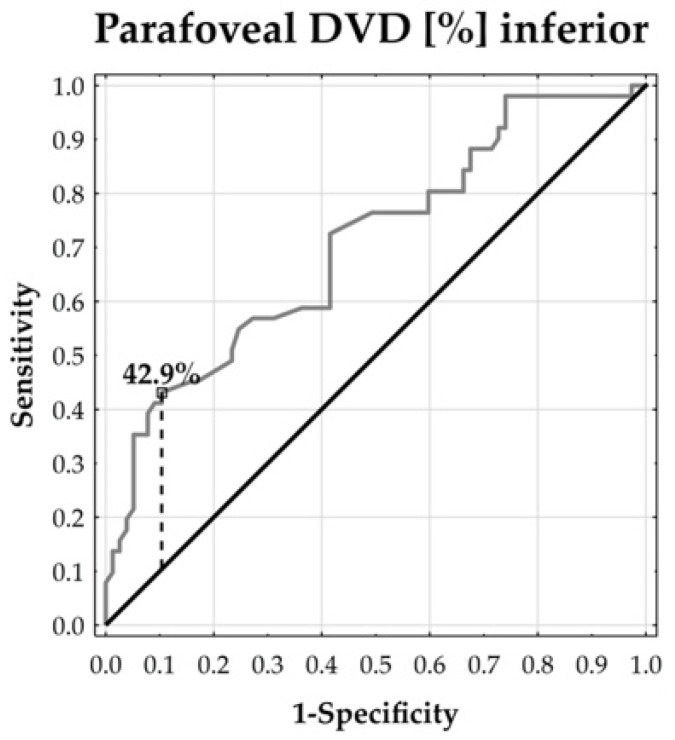
ROC curve for inferior DVD. AUC = 70.47% (*p* < 0.0001).

The ROC curves demonstrated that the parafoveal DVD could distinguish between the patients and healthy subjects. The temporal and inferior parafoveal DVDs revealed the largest separability, reflected by the corresponding highest area under the curve (AUC: 73.57% and 70.47%, respectively, both at *p* < 0.0001). In addition, the temporal parafoveal DVD’s sensitivity was 47.06% and its specificity reached 94.81%. The inferior parafoveal DVD’s sensitivity was 43.14% and its specificity was 86.61%. This means that the temporal and inferior DVDs tended to enable the accurate specification of true negative cases, whereas the identification of true positive cases remained moderate (Figure 2, Figure 3, Figure 4, Figure 5 and Figure 6).

We compared the OCTA parameters, i.e., the superficial FAZ, deep FAZ, parafoveal mean SVD, and parafoveal mean DVD, to the capillaroscopy findings and scleroderma parameters (Table 4, Table 5, Table 6 and Table 7).

Patients with an early scleroderma pattern in capillaroscopy showed a larger superficial FAZ than those with active and late patterns (*p* = 0.0104).

There was also a statistically significant difference when comparing the FAZ in the early to active (*p* < 0.0001) and early to late scleroderma patterns (*p* < 0.0001). However, there was no statistically significant difference in the comparison between the active and late phases.

Moreover, the superficial FAZ was larger among patients without hemorrhages (*p* = 0.0198). Similar results were observed in the deep FAZ according to the scleroderma pattern in capillaroscopy (*p* = 0.0076) and in the presence of hemorrhages (*p* = 0.0152). A statistically significant difference was found regarding interstitial lung diseases, i.e., NSIP and UIP, in reference to the deep FAZ area (*p* = 0.0484). SSc patients with NSIP had a larger FAZ than UIP patients (*p* = 0.0484). Moreover, NSIP subjects had a higher parafoveal mean superficial vessel density than UIP patients (*p* = 0.471). Furthermore, patients undergoing sildenafil treatment demonstrated a decreased FAZ in both the superficial and deep capillary plexuses (*p* = 0.0178, *p* = 0.0222, respectively) (Table 4 and Table 5).

## 4. Discussion

Vasculopathy is the main and initial step in the pathogenesis of SSc and is not only observed in the skin but also in the internal organs [3]. The vascular injury, followed by impaired neovascularization and vascular remodeling, leads to capillary dilation, stenosis of the arterioles, and the loss of small capillaries [18]. The retinal circulatory system, as one of the most active tissues in the body, is susceptible to injury. Microvasculatory changes in SSc mostly affect the arterioles and small capillaries; therefore, the retinal microvasculature seems to be ideal for disease observation and early change detection. 

Some previous studies have revealed retinal findings in SSc patients. Ushiyama et al. demonstrated a higher incidence of retinal pathologies compared to healthy controls (*p* = 0.011), consisting of hard exudates, vascular tortuosity, microhemorrhages, and macular degeneration [19]. In addition, Gomes et al. observed retinal microvascular abnormalities including arteriolar narrowing, vascular tortuosity, and arteriovenous nicking [14]

In our study, we used OCTA to examine the retinal microvasculature in patients with SSc and in a healthy group. 

The FAZ is the central vessel-free area of the macula, surrounded by a network of capillaries. Studies suggest that systemic diseases correlate with the FAZ size. Kok et al. revealed a decreased FAZ area in Ssc patients [20]. In contrast, no statistically significant differences were observed in comparison to healthy subjects regarding the FAZ size in other studies conducted by Hekimsoy et al. [21], Mihalovic et al. [22], and Cutolo et al. [23]. Their findings were similar to ours.

Interestingly, we discovered an increased parafoveal deep vessel density in SSc patients compared to controls. Scleroderma microangiopathy includes reduced normal vessels, avascular areas, and capillary neovascularization, leading to the formation of enlarged and bushy capillaries, as observed in NC [24]. Carnevli et al. [25] revealed abnormal capillaries in the OCTA images of SSc patients, such as megacapillaries, as found in NC. These pathologic giant capillaries and branched vessels might be detected by OCTA and increase the vessel density. However, in most studies, the authors have demonstrated decreased percentages in VD. Hekimsoy et al. [21] revealed a lower superficial vessel density and deep vessel density in the fovea in patients compared to the control group. According to the DVD, similar results were observed by Carnevli et al. [25]. Rothe et al. demonstrated a reduced macular VD in the superficial capillary plexus [26]. Kok et al. demonstrated a reduced capillary density in both the deep and superficial plexuses among SSc patients with retinopathy [20].

The presence of characteristic hallmarks observed in peripheral capillaroscopy correlate with internal organ involvement, e.g., the lungs and kidneys. Furthermore, the changes observed in NC are considered to reflect the stages of microangiopathy [27].

Ushiyama et al. compared the nailfold capillary findings of SSc patients with the retinal findings of SSc patients without them, and the results showed no significant correlation between the two groups [19]. In contrast, our study showed a significant correlation between the nailfold capillaroscopy and OCTA findings among SSc patients. Firstly, the superficial and the deep FAZ areas were correlated with the scleroderma patterns. Our study demonstrated enlarged superficial and deep areas in the early phase of the disease and decreased areas in the late phase. An early pattern is defined as a few giant capillaries, the presence of hemorrhages without the loss of capillaries, and normal capillary perfusion [28]. As the disease progresses, we observe extensive avascular areas, branched capillaries, and the disorganization of the normal capillary architecture [28]. An enlarged FAZ in the early stages of the disease could suggest the dominance of the ischemic and atrophic stages, as well as in the retinal microcirculatory system. Furthermore, we observed a decreased FAZ area in the late pattern due to the presence of abnormal, branched vessels detected by OCTA. To our knowledge, this is the first study that shows FAZ area changes in relation to the stages of NC. Consequently, hemorrhages, characteristic of the early phase of SSc, correlated positively with an increased superficial FAZ.

However, we did not observe a statistically significant correlation between avascular areas, giant capillaries, and branched vessels as detected via the NC and OCTA parameters. 

Mihailovic et al., in their study, revealed a significant correlation between the nailfold capillary density and the VD of the choriocapillaris [22]. However, we did not find a correlation between the vessel density and the scleroderma pattern or number of vessels in NC. 

Other studies have analyzed OCTA parameters with pulmonary tests in SSc patients. Cerasuolo et al. detected a correlation between chorioretinal perfusion and the diffusion capacity of the lungs for carbon monoxide (DLCO) in scleroderma patients [18].

However, Cutolo et al. did not find a significant difference in the OCTA parameters between SSc patients with interstitial lung disease (ILD) and those without it [23]. Our results showed a significant correlation between the type of lung disease, the FAZ size, and the parafoveal superficial vessel density. SSc patients with nonspecific interstitial pneumonia (NSIP) had a larger FAZ than those with usual interstitial pneumonia (UIP) (*p* = 0.0484). Moreover, NSIP cases had a higher parafoveal mean superficial vessel density than those with UIP (*p* = 0.0471). 

Moreover, we studied the effects of vasodilator drugs used to manage the consequences of scleroderma vasculopathy, such as digital ulcers, Raynaud’s syndrome, and pulmonary hypertension. We observed a decreased FAZ in both the superficial and deep capillary plexuses in those treated with sildenafil when compared to the SSc group without vasodilator therapy. However, we did not find a significant difference in the OCTA parameters in patients treated with amlodipine. These results indicate that vasodilator therapy can influence the OCTA parameters, reducing the suspected ischemic effect and FAZ enlargement. Polak et al. [29], in their study, investigated the effects of sildenafil on the retinal blood flow in 12 healthy male patients. Their results showed increased retinal blood flow compared to the placebo group (*p* = 0.029) and no effect on vasodilatation in the retinal arteries or veins. 

In our analysis, we did not find significant differences in the OCTA parameters according to hypertension or the immunosuppressive treatment method. 

Our study demonstrates the correlation between the peripheral microvascular system and ocular microcirculatory impairment. The strengths of the study included the size of the rheumatological dataset. We also acknowledge the limitations of the research, which include the limited number of participants and the cross-sectional nature of the study. 

Nonetheless, our study is the first to reveal a significant correlation between the stage of the disease according to the NC findings and the OCTA parameters.

## 5. Conclusions

Our investigation showed that the peripheral microvascular system is correlated with ocular microcirculatory impairment. OCTA is a useful tool for the assessment of microvascular impairment in scleroderma patients. Based on our study, we suggest regular ophthalmic examination in SSc patients for the early detection and management of ocular manifestations. 

However, further studies are needed on larger numbers of patients to corroborate our results.

## Figures and Tables

**Table 1:** Baseline characteristics of the study participants (*n* = 72).

Trait	*n* M (SD) *	% Me (Q_1_–Q_3_) **
Gender:		
- Female	49	68.1
- Male	23	31.9
Age (year)	41.3 (11.2)	40.0 (32.0–53.0)
Scleroderma	28	38.9

* For discrete variables: *n*—number; %—percentage. ** For numerical traits: M—mean; SD—standard deviation; Me—median; Q—quartile.

**Table 2:** Baseline characteristics of the scleroderma group (*n* = 31).

Trait	*n* M (SD)	% Me (Q_1_–Q_3_)
Gender:		
- Female	22	71.0
- Male	9	29.0
Scleroderma:		
- Localized	8	28.6
- Systemic	20	71.4
Capillaroscopy	27	96.4
Scleroderma pattern:		
- None	2	7.1
- Early	5	17.9
- Active	12	42.9
- Late	9	32.1
PM/SCL	1	3.6
PC TH fibrillarin	3	10.7
SCI70	17	54.8
Centromere	8	25.8
Number of vessels:		
- Normal	2	7.1
- Reduced	20	71.5
- Very reduced	6	21.4
Avascular area	16	57.1
Giant capillaries	14	50.0
Hemorrhages	11	39.3
Branched vessels	10	35.7
MES	2.2 (1.1)	2.5 (1.1–3.1)
Interstitial lung disease	12	42.9
PAH-1	2	7.1
NSIP	7	22.6
UIP	4	14.3
MMF	13	41.9
MTX	7	25.0
mRSS	8.1 (7.9)	2.0 (2.0–12.0)
Finger ulcers	8	28.6
RVSP > 35 mmHg	3	1.1
Elevated proBNP	5	18.5
Arterial hypertension	8	25.8
Administration of sildenafil	13	41.9
Administration of amlodipine	12	38.7

Missing data were pair-wise deleted. For discrete variables: *n*—number; %—percentage. For numerical traits: M—mean; SD—standard deviation; Me—median; Q—quartile.

**Table 3:** Descriptive statistics for the selected numerical measures of the study participants’ eyes.

Investigated Trait	Cases (*n* = 61 Eyes)		Controls (*n* = 81 Eyes)		*p*-Value **
	M (SD) *	Me (Q_1_–Q_3_) *	M (SD) *	Me (Q_1_–Q_3_) *	
Age (years)	46.5 (9.7)	48 (40–54)	44.1 (9.6)	41 (35–55)	=0.1520
FAZ superficial	0.260 (0.104)	0.26 (0.17–0.35)	0.246 (0.095)	0.25 (0.18–0.30)	=0.5361
FAZ deep	0.248 (0.176)	0.18 (0.12–0.37)	0.202 (0.147)	0.16 (0.12–0.24)	=0.1527
Foveal SVD	23.3 (5.5)	23.0 (12.3–38.6)	24.0 (3.9)	23.5 (22.0–26.5)	=0.3767
Parafoveal SVD superior	41.1 (2.9)	41.1 (19.5–26.6)	41.7 (1.6)	41.8 (41.0–42.7)	=0.0655
Parafoveal SVD inferior	41.9 (2.0)	41.8 (40.2–42.5)	42.0 (1.1)	41.9 (41.1–42.7)	=0.5463
Parafoveal SVD temporal	39.1 (3.5)	39.2 (37.6–41.1)	39.7 (1.8)	39.5 (38.5–40.5)	=0.0705
Parafoveal SVD nasal	40.1 (3.1)	40.3 (38.6–41.5)	40.6 (1.7)	40.5 (39.6–41.8)	=0.1247
Parafoveal mean SVD	40.6 (2.5)	40.3 (39.7–41.6)	41.0 (1.3)	40.8 (40.2–41.8)	=0.0713
Foveal DVD	27.4 (7.6)	29.6 (23.2–32.5)	28.0 (9.0)	32.4 (21.4–35.0)	=0.8505
Parafoveal DVD superior	**41.8 (2.7)**	**42.1 (41.0–43.6)**	**38.9 (5.0)**	**41.3 (32.8–42.1)**	**<0** **.** **0001**
Parafoveal DVD inferior	**42.0 (3.1)**	**42.3 (41.4–43.8)**	**38.8 (5.3)**	**41.3 (32.3–42.1)**	**<0.0001**
Parafoveal DVD temporal	**42.1 (2.6)**	**42.6 (40.5–44.3)**	**38.2 (5.3)**	**40.8 (32.5–41.9)**	**<0.0001**
Parafoveal DVD nasal	**42.0 (3.7)**	**42.6 (41.2–44.1)**	**39.2 (5.7)**	**41.7 (31.8–42.9)**	**<0.0001**
Mean parafoveal DVD	**42.0 (2.5)**	**42.1 (41.1–43.8)**	**38.8 (5.3)**	**41.4 (32.1–42.0)**	**<0.0001**

* M—mean; Me—median; SD—standard deviation; minimum-to-maximum values. ** A multifactor model, controlled for age and gender, was implemented. Bold values indicate statistical significance.

**Table 4:** Descriptive statistics for the superficial FAZ in the study participants by selected clinical conditions (*n* = 28).

Investigated Trait	Statistical Parameter		*p*-Value
	M (SD)	Me (Q_1_–Q_3_)	
Localized scleroderma	0.271 (0.084)	0.270 (0.240–0.345)	=0.6331
Systemic scleroderma	0.255 (0.115)	0.250 (0.150–0.330)	
Scleroderma pattern:			**=0.0104**
● Early	0.399 (0.073)	0.390 (0.350–0.445)	
● Active	0.239 (0.088)	0.250 (0.150–0.300)	
● Late *	0.222 (0.089)	0.230 (0.140–0.280)	
SCI70	0.256 (0.121)	0.245 (0.145–0.350)	=0.6184
Centromere	0.271 (0.084)	0.270 (0.240–0.345)	
Number of vessels:			=0.4979
● Normal	0.322 (0.080)	0.315 (0.255–0.390)	
● Reduced	0.260 (0.112)	0.260 (0.160–0.345)	
● Very reduced	0.237 (0.080)	0.240 (0.220–0.280)	
Avascular areas:			=0.7091
● Yes	0.255 (0.100)	0.275 (0.155–0.340)	
● No	0.267 (0.111)	0.250 (0.180–0.340)	
Giant capillaries:			=0.1753
● Yes	0.239 (0.086)	0.250 (0.150–0.300)	
● No	0.282 (0.117)	0.270 (0.180–0.380)	
Hemorrhages:			**=0.0198**
● Yes	0.216 (0.087)	0.225 (0.135–0.280)	
● No	0.285 (0.106)	0.270 (0.230–0.350)	
Branched vessels:			=0.7631
● Yes	0.268 (0.100)	0.270 (0.180–0.350)	
● No	0.257 (0.107)	0.250 (0.175–0.320)	
Interstitial lung disease:			=0.9067
● Yes	0.258 (0.105)	0.270 (0.150–0.350)	
● No	0.262 (0.106)	0.250 (0.180–0.340)	
NSIP	0.286 (0.102)	0.270 (0.230–0.380)	=0.1248
UIP	0.185 (0.101)	0.155 (0.120–0.250)	
MMF	0.286 (0.118)	0.270 (0.210–0.380)	=0.4313
MTX	0.238 (0.095)	0.250 (0.140–0.300)	
Finger ulcers:			=0.4087
● Yes	0.241 (0.105)	0.250 (0.140–0.330)	
● No	0.269 (0.106)	0.255 (0.180–0.350)	
Arterial hypertension:			=0.5162
● Yes	0.242 (0.092)	0.245 (0.140–0.340)	
● No	0.267 (0.111)	0.270 (0.180–0.350)	
Administration of sildenafil:			**=0.0178**
● Yes	0.218 (0.098)	0.215 (0.140–0.300)	
● No	0.292 (0.103)	0.280 (0.240–0.350)	
Administration of amlodipine:			=0.0626
● Yes	0.292 (0.121)	0.280 (0.180–0.380)	
● No	0.231 (0.083)	0.240 (0.170–0.300)	

M—mean; SD—standard deviation; Me—median; Q—quartile. * Scleroderma pattern, post-hoc multiple comparisons: early vs. active *p* < 0.0001; early vs. late *p* < 0.0001; active vs. late NS. Bold values indicate statistical significance.

**Table 5:** Descriptive statistics for the deep FAZ (%) in the study participants by selected clinical conditions (*n* = 28).

Investigated Trait	Statistical Parameter		*p*-Value
	M (SD)	Me (Q_1_–Q_3_)	
Localized scleroderma	0.237 (0.171)	0.190 (0.125–0.240)	=0.9996
Systemic scleroderma	0.237 (0.152)	0.180 (0.110–0.370)	
Scleroderma pattern:			**=0.0076**
● Early	0.456 (0.142)	0.425 (0.345–0.555)	
● Active	0.197 (0.119)	0.160 (0.110–0.260)	
● Late *	0.173 (0.132)	0.110 (0.100–0.330)	
SCI70	0.243 (0.163)	0.180 (0.100–0.390)	=0.9997
Centromere	0.237 (0.171)	0.190 (0.125–0.240)	
Number of vessels:			=0.1350
● Normal	0.435 (0.252)	0.410 (0.220–0.650)	
● Reduced	0.227 (0.140)	0.185 (0.110–0.335)	
● Very reduced	0.182 (0.114)	0.120 (0.110–0.290)	
Avascular areas:			=0.2698
● Yes	0.214 (0.138)	0.120 (0.105–0.350)	
● No	0.263 (0.177)	0.190 (0.150–0.260)	
Giant capillaries:			=0.2396
● Yes	0.210 (0.120)	0.180 (0.120–0.290)	
● No	0.262 (0.185)	0.210 (0.110–0.390)	
Hemorrhages:			**=0.0152**
● Yes	0.174 (0.079)	0.160 (0.115–0.240)	
● No	0.271 (0.179)	0.220 (0.110–0.390)	
Branched vessels:			=0.4219
● Yes	0.267 (0.135)	0.300 (0.150–0.390)	
● No	0.225 (0.166)	0.180 (0.110–0.275)	
Interstitial lung disease:			=0.3505
● Yes	0.270 (0.150)	0.315 (0.100–0.390)	
● No	0.222 (0.160)	0.170 (0.110–0.260)	
NSIP	0.324 (0.142)	0.370 (0.330–0.390)	**=0.0484**
UIP	0.140 (0.112)	0.110 (0.070–0.210)	
MMF	0.247 (0.147)	0.190 (0.120–0.390)	=0.3844
MTX	0.193 (0.109)	0.190 (0.110–0.220)	
Finger ulcers:			=0.3582
● Yes	0.207 (0.129)	0.135 (0.110–0.330)	
● No	0.249 (0.171)	0.190 (0.110–0.370)	
Arterial hypertension:			=0.2474
● Yes	0.195 (0.119)	0.150 (0.120–0.260)	
● No	0.251 (0.171)	0.190 (0.110–0.370)	
Administration of sildenafil:			**=0.0222**
● Yes	0.181 (0.125)	0.115 (0.100–0.300)	
● No	0.279 (0.173)	0.210 (0.150–0.420)	
Administration of amlodipine:			=0.0780
● Yes	0.278 (0.177)	0.210 (0.120–0.390)	
● No	0.200 (0.137)	0.140 (0.100–0.300)	

M—mean; SD—standard deviation; Me—median; Q—quartile. * Scleroderma pattern, post-hoc multiple comparisons: early vs. active *p* < 0.0001; early vs. late *p* < 0.0001; active vs. late NS. Bold values indicate statistical significance.

**Table 6:** Descriptive statistics for the parafoveal mean SVD (%) in the study participants by selected clinical conditions (*n* = 28).

Investigated Trait	Statistical Parameter		*p*-Value
	M (SD)	Me (Q_1_–Q_3_)	
Localized scleroderma	40.68 (2.25)	40.01 (39.36–42.47)	=0.6807
Systemic scleroderma	40.93 (1.95)	40.35 (39.95–41.62)	
Scleroderma pattern:			=0.6174
● Early	41.57 (1.79)	40.97 (40.29–42.88)	
● Active	40.86 (2.24)	40.22 (39.70–43.25)	
● Late	40.37 (2.12)	40.22 (38.77–41.63)	
SCI70	41.10 (2.07)	40.46 (40.04–42.63)	=0.6908
Centromere	40.68 (2.25)	40.01 (39.36–42.48)	
Number of vessels:			=0.1146
● Normal	42.83 (1.75)	40.94 (41.34–44.33)	
● Reduced	40.65 (1.97)	40.29 (39.36–41.63)	
● Very reduced	40.64 (2.12)	40.22 (39.75–40.35)	
Avascular areas:			=0.3032
● Yes	40.56 (1.97)	40.26 (39.72–41.60)	
● No	41.17 (2.12)	40.97 (39.75–43.25)	
Giant capillaries:			=0.7616
● Yes	40.93 (2.24)	40.59 (39.70–43.25)	
● No	40.75 (1.88)	40.35 (39.75–41.63)	
Hemorrhages:			=0.1405
● Yes	40.26 (1.81)	40.17(39.55–41.31)	
● No	41.16 (2.12)	40.35 (39.85–43.63)	
Branched vessels:			=0.5786
● Yes	41.10 (1.40)	40.57 (40.30–41.63)	
● No	40.74 (2.26)	40.22 (39.36–42.48)	
Interstitial lung disease:			=0.2209
● Yes	41.38 (1.88)	41.07 (40.22–43.63)	
● No	40.60 (2.09)	40.22 (39.40–41.63)	
NSIP	42.21 (1.69)	41.62 (40.57–43.80)	**=0.0471**
UIP	39.81 (1.40)	39.54 (38.69–40.94)	
MMF	40.76 (1.51)	40.35 (39.95–41.58)	=0.8716
MTX	41.10 (2.43)	40.97 (39.70–43.25)	
Finger ulcers:			=0.3372
● Yes	40.43 (2.00)	40.32 (39.70–41.05)	
● No	41.05 (2.10)	40.35 (39.75–43.25)	
Arterial hypertension:			=0.7358
● Yes	41.02 (2.07)	40.32 (39.70–41.63)	
● No	40.76 (2.12)	40.22 (39.75–41.63)	
Administration of sildenafil:			=0.3159
● Yes	40.44 (1.79)	40.32 (39.75–41.05)	
● No	41.09 (2.27)	40.22 (39.70–43.38)	
Administration of amlodipine:			=0.9567
● Yes	40.80 (1.77)	40.30 (39.80–41.63)	
● No	40.84 (2.39)	40.29 (39.22–43.63)	

M—mean; SD—standard deviation; Me—median; Q—quartile. Bold values indicate statistical significance.

**Table 7:** Descriptive statistics for the parafoveal mean DVD (%) in the study participants by selected clinical conditions (*n* = 28).

Investigated Trait	Statistical Parameter		*p*-Value
	M (SD)	Me (Q_1_–Q_3_)	
Localized scleroderma	42.55 (1.44)	42.34 (41.46–43.93)	=0.6199
Systemic scleroderma	42.21 (2.47)	42.12 (41.15–43.90)	
Scleroderma pattern:			=0.4437
● Early	41.57 (4.02)	43.54 (39.21–44.06)	
● Active	42.51 (1.50)	42.04 (41.47–43.80)	
● Late	42.08 (1.70)	41.22 (41.07–44.38)	
SCI70	42.01 (2.64)	42.04 (40.94–44.09)	=0.6192
Centromere	42.55 (1.44)	42.34 (41.46–43.93)	
Number of vessels:			=0.3402
Normal	43.84 (0.52)	43.84 (43.40–44.29)	
Reduced	42.24 (2.35)	42.44 (41.36–43.85)	
Very reduced	41.99 (1.60)	41.47 (41.22–41.63)	
Avascular areas:			=0.7687
● Yes	42.42 (1.49)	41.76 (41.22–43.75)	
● No	42.23 (2.76)	43.05 (41.45–44.25)	
Giant capillaries:			=0.3396
● Yes	42.65 (1.51)	42.44 (41.47–43.90)	
● No	42.03 (2.62)	42.00 (41.15–44.23)	
Hemorrhages:			=0.8356
● Yes	42.42 (1.23)	42.10 (41.49–43.55)	
● No	42.28 (2.54)	43.05 (41.22–44.35)	
Branched vessels:			=0.1093
● Yes	43.14 (1.32)	43.38 (42.12–43.90)	
● No	42.00 (2.34)	41.69 (41.19–43.93)	
Interstitial lung disease:			=0.2237
● Yes	42.92 (1.73)	43.54 (41.07–44.38)	
● No	42.07 (2.29)	42.00 (41.27–43.63)	
NSIP	43.58 (1.50)	43.80 (43.37–44.45)	=0.1244
UIP	41.46 (1.66)	40.82 (40.44–42.49)	
MMF	41.88 (2.79)	42.13 (41.22–43.70)	=0.5305
MTX	42.70 (1.63)	43.35 (41.47–44.25)	
Finger ulcers:			=0.8485
● Yes	42.42 (1.54)	42.10 (41.07–43.70)	
● No	42.28 (2.43)	42.47 (41.27–44.25)	
Arterial hypertension:			=0.9677
● Yes	42.31 (1.78)	41.61 (41.45–44.45)	
● No	42.28 (2.32)	41.12 (41.22–43.90)	
Administration of sildenafil:			=0.8966
● Yes	42.23 (1.45)	41.94 (41.07–43.48)	
● No	42.32 (2.62)	42.95 (41.47–44.35)	
Administration of amlodipine:			=0.3429
● Yes	41.95 (2.64)	41.72 (41.47–44.23)	
● No	42.59 (1.64)	42.44 (41.15–43.90)	

M—mean; SD—standard deviation; Me—median; Q—quartile.

## Data Availability

The data presented in this study are available on request from the corresponding authors.
